# Concentrations of selected immunological parameters in the serum and processing fluid of suckling piglets and the serum and colostrum of their mothers

**DOI:** 10.1186/s12917-024-04024-9

**Published:** 2024-05-03

**Authors:** Agata Augustyniak, Ewelina Czyżewska-Dors, Małgorzata Pomorska-Mól

**Affiliations:** 1 Department of Preclinical Sciences and Infectious Diseases, Poznan University of Life Sciences, Wołyńska 35, Poznań, 60-637 Poland; 2 Department of Internal Diseases and Diagnostics, Poznan University of Life Sciences, Wołyńska 35, Poznań, 60- 637 Poland

**Keywords:** Pigs, Neonate, Immunoglobulins, Cytokines, Acute phase proteins, Matrices, Processing fluid

## Abstract

**Background:**

Blood sampling from neonatal piglets is related to multiple disadvantages. Therefore, a new, alternative matrix is required to assess piglets’ early immune status efficiently. The present study aimed to assess the usefulness of processing fluid for determining selected piglets’ immune parameters. 264 pigs − 31 sows, 146 male piglets, and 87 female piglets from commercial indoor farrow-to-finish pig herd were included in this study. 264 serum, 31 colostrum, and 146 processing fluid samples were collected. Serum was collected from all animals, colostrum was collected from sows, and processing fluid was collected from male piglets only. Using commercial ELISA tests, the concentration of various immunoglobulins, cytokines, and acute phase proteins was assessed in each matrix. Statistical analyses were employed to determine differences in the concentration of measured indices between piglets’ serum and processing fluid and correlations in the concentration of tested indices between particular sets of matrices.

**Results:**

Statistical analyses did not reveal significant differences in the IgG, IgA, IL-1β, IL-4, IL-6, and IFN-γ concentration between piglets’ serum and processing fluid (*p* > 0.05). A positive correlation (*p* < 0.05) regarding the concentration of some indices between processing fluid and samples collected from sows was also observed.

**Conclusions:**

Processing fluid can be considered a promising alternative to blood for assessing some immunological indices in piglets, such as IgG, IgA, IL-1β, IL-4, IL-6, and IFN-γ, and, possibly, in the indirect assessment of some indices in lactating sows, including IgA, IL-1β, IL-4, IL-6, IL-8, IFN-γ, or Pig-MAP.

## Background

Blood and serum are the most common matrices for monitoring the pigs’ health status. However, blood collection from newborn piglets is a labour-intensive and time-consuming method [1]. Moreover, it is stressful for piglets and may negatively affect their welfare [1]. Importantly, blood sampling from piglets, especially during their first days of life, carries a high mortality risk [1]. Due to these reasons, early monitoring is often postponed until the later stages of piglet life. In consequence, detecting possible disorders can be delayed. For example, inadequate colostrum intake is one of the significant reasons for neonatal piglets’ mortality and may negatively influence piglets’ weight gain [2, 3].

Given the above and the importance of piglets’ immune status for further development and health, identifying samples that are easy to collect during the first day of life and cost-effective is desirable. Examples of such alternative matrices are oral fluid (OF) and processing fluid (PF). OF has already been approved for the detection of antibodies and pathogen genetic material in swine [4]. However, its utility in piglets is doubtful, as OF collection from pigs younger than one week is almost impossible. In this case, PF seems to be a much more promising alternative. PF usually consists of blood and tissue fluids recovered from castrated testicles and docked tails during piglet processing (3–5 days of age) [5]. The abovementioned processing procedures are routinely performed on many pig farms worldwide [6]. Thus, collecting such samples does not require additional effort and does not generate excessive stress for piglets. The available studies indicate that PF can be a promising, practical, and inexpensive sample that improves the herd monitoring of, among others, PRRS or PCV2 [7, 8]. Therefore, it is reasonable to assume that PF can be a practical tool for other purposes, such as assessing piglets’ immune components.

It is hypothesised that PF can be used as an alternative sample to serum for the early assessment of porcine immune status. There is no data concerning the utility of PF as the matrix to evaluate immunological parameters such as immunoglobulins (Ig), cytokines, or acute phase proteins (APP). Therefore, the objective of the current study was to evaluate the usefulness of PF compared to serum for the detection of Ig (IgG, IgA, IgM), cytokines (IL-1β, IL-4, IL-6, IL-8, INF-γ, and TNF-α) and APP (haptoglobin (Hp), C-reactive protein (CRP), pig major acute phase protein (Pig-MAP) and serum amyloid A (SAA)). In addition, the correlation between the concentration of tested parameters in samples collected from sows and their litters was investigated.

## Results

### Immunoglobulins

**Fig. 1:**
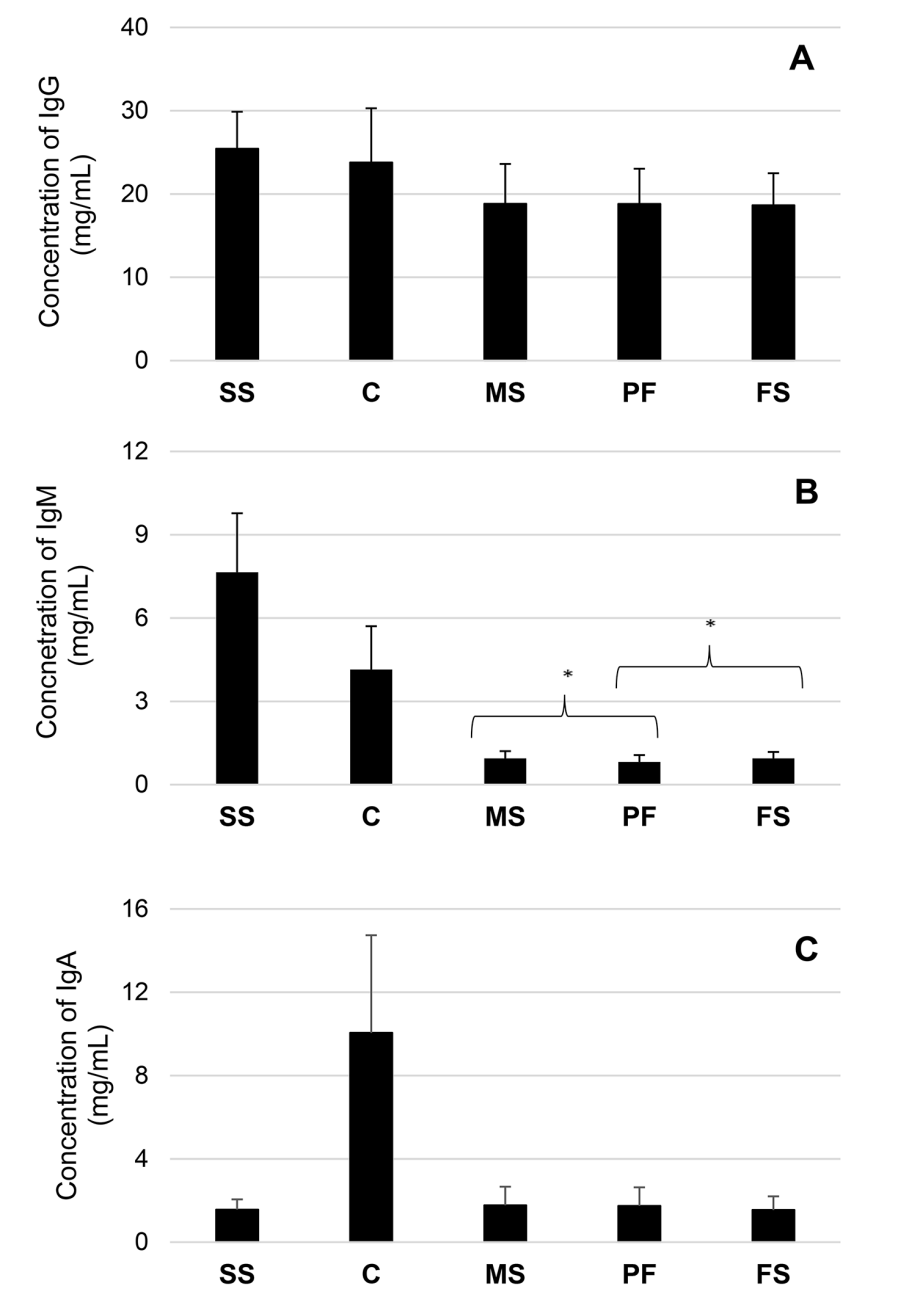
(**A-C**) The mean concentration (± SD) (mg/ml) of immunoglobulins: IgG, IgM, IgA in tested samples. SS – sows serum, C – colostrum, MS – male piglets’ serum, PF – processing fluid, FS – female piglets’ serum

All tested Ig classes were detected in analysed matrices (concentration range for sows’ serum: 16.6-36.46, 4.29–12.64, and 0.89–2.65, for male piglets’ serum: 9.6-32.84, 0.45–1.7, and 0.71–3.99, for female piglets’ serum: 10.02–29.66, 0.57–1.69, and 0.71–3.17, for PF: 2.56–31.28, 0.39–1.94, and 0.61–4.35, for colostrum: 12.4-39.27, 2.45–10.34, and 3.1–18.8, respectively for IgG, IgM and IgA, all values in mg/mL). IgG reached the highest concentration in all tested samples (Fig. 1). No significant differences were found between IgG content in PF and male piglets’ serum (*p* = 0.77), PF and female piglets’ serum (*p* = 0.57), and male and female piglets’ serum (*p* = 0.78; Fig. 1). A weak positive correlation was detected only between the IgG concentration in sows’ sera and female piglets’ sera (Table 1).

The concentration of IgA in all tested matrices was second to IgG, except for sows’ serum in which IgA was the least concentrated one (Fig. 1). No significant differences between the concentration of IgA in the PF and the sera of male (*p* = 0.93) and female (*p* = 0.15) piglets were observed (Fig. 1C). No differences were identified between IgA levels in the serum of male and female piglets (*p* = 0.78; Fig. 1C). A weak but statistically significant correlation between the IgA concentration in sows’ serum and colostrum was observed (Table 1). Moreover, the concentration of IgA in sows’ serum was positively correlated with its concentration in PF and female piglets’ sera (Table 1). No statistically significant correlation was observed regarding the IgA concentration in the rest of the analysed matrices (Table 1).

The second most concentrated Ig in the sera collected from sows was IgM (Fig. 1B). In the remaining matrices, the concentration of IgM was, however, exceeded not only by the level of IgG but also by IgA (Fig. 1). The piglets’ serum of both genders showed similar amounts of IgM (*p* = 0.83). However, significant differences were detected between PF and sera of male (*p* = 0.00001) and female (*p* = 0.00001) piglets (Fig. 1B). No significant correlations between concentrations of IgM in different matrices were found (*p* > 0.05; Table 1).

**Table 1:** The correlations (R-Spearman coefficient) between tested matrices in the IgG, IgM, and IgA concentrations. Brackets contain *p*-values for each correlation

	Immunoglobulins					
	IgG		IgA		IgM	
Matrice	Sows’ serum	Colostrum	Sows’ serum	Colostrum	Sows’ serum	Colostrum
Colostrum	-0.13 (*p* = 0.49)	1	0.39* (*p* = 0.03)	1	0.04 (*p* = 0.83)	1
Male piglets’ serum	0.02 (*p* = 0.85)	0.14 (*p* = 0.09)	0.14 (*p* = 0.09)	-0.12 (*p* = 0.16)	-0.00 (*p* = 0.98)	0.05 (*p* = 0.57)
Processing fluid	-0.08 (*p* = 0.35)	-0.04 (*p* = 0.62)	0.25* (*p* = 0.00)	-0.10 (*p* = 0.23)	-0.08 (*p* = 0.31)	0.04 (*p* = 0.59)
Female piglets’ serum	0.23* (*p* = 0.03)	0.16 (*p* = 0.13)	0.54* (*p* = 0.00)	-0.12 (*p* = 0.05)	-0.06 (*p* = 0.59)	0.04 (*p* = 0.70)

*Correlation is significant (*p* < 0.05)

### Cytokines

**Fig. 2:**
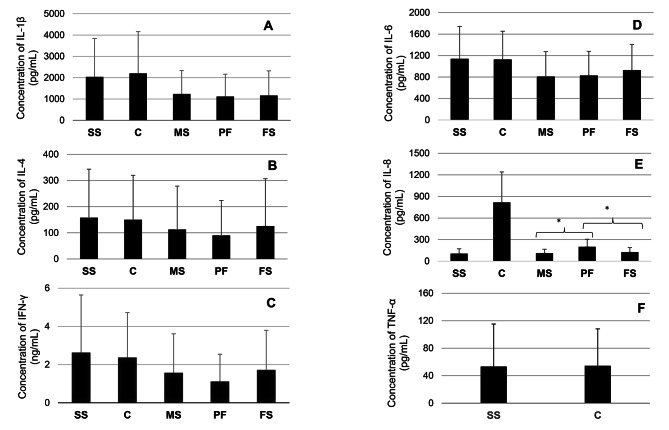
(**A-F**) The mean concentration (± SD) (mg/ml) of cytokines: IL-1β, IL-4, IFN-γ, IL-6, IL-8, TNF – α in tested samples. SS – sows serum, C – colostrum, MS – male piglets’ serum, PF – processing fluid, FS – female piglets’ serum

The presence of IL-1β, IL-4, IL-6, IL-8, and INF-γ was confirmed in all types of tested matrices (concentration range of IL-1β (pg/mL) was as follows: for sows’ serum: 310.02–6895.00, for male piglets’ serum: 122.01–4674.00, for female piglets’ serum: 127.3-5234.6, for PF: 192.73-4691.2, for colostrum: 397.37–7976.00. The concentration range of IL-4 (pg/mL) was as follows: for sows’ serum: 5.22–567.4, for male piglets’ serum: 1.5–578.00, for female piglets’ serum: 1.5-601.4, for PF: 1.59–569.00, for colostrum: 7.75–506.2. For IL-6 (pg/mL), the minimum and maximum concertation were equal: for sows’ serum 332.66-2310.7, for male piglets’ serum: 56.8-2266.4, for female piglets’ serum 82.67-2378.4, for PF 67.5-2398.2 for colostrum 289.32-2247.4, IL-8 (pg/mL) for sows’ serum 25.96-301.12, for male piglets’ serum: 23.24-319.33, for female piglets’ serum 21.07-316.83, for PF 42.29-573.14, for colostrum 333.4-1964.98, for INF- γ (ng/mL) for sows’ serum 0.59–13.65, for male piglets’ serum: 0.5-11.18, for female piglets’ serum 0.5-10.57; for PF 0.5–10.5; for colostrum 0.54–9.51. TNF-α was detected only in sows’ serum (range: 28–210 pg/ml) and colostrum (range 22–360 pg/ml). Out of all tested cytokines, IL-1β was the most abundant cytokine in the PF (Fig. 2A). In the remaining types of samples, IL-1β concentration was second to IFN-γ only (Fig. 2). No significant differences in the IL-1β concentration in PF and sera of male (*p* = 0.20) and female (*p* = 0.73) piglets were demonstrated (*p* > 0.05; Fig. 2A). Differences were also not identified between IL-1β concentrations in piglet sera of both genders (*p* = 0.19). IL-1β concentrations were correlated across multiple matrices (Table 2).

The mean concentration of IL-4 prevailed over IL-8 in the sera of all analysed groups (Fig. 2). In the colostrum and PF, its concentration was lower compared to IL-8 (Fig. 2). Similarly to the previously described parameter, no significant differences between the concentration of IL-4 in PF and the sera of male (*p* = 0.59) and female (*p* = 0.41) piglets were observed (Fig. 2B). IL-4 concentration was also similar in the male and female piglets’ sera (*p* = 0.73; Fig. 2B). Significant correlations between the concentration of IL-4 were determined between all tested sets of matrices (Table 2).

IL-6 was the third most concentrated cytokine in each kind of matrix (Fig. 2). Its concentration in PF and sera of male (*p* = 0.55) and female (*p* = 0.14) piglets did not differ significantly (Fig. 2D). The level of IL-6 in the male and female piglets’ sera was also similar (*p* = 0.07). Significant correlations in the concentration of IL-6 were determined between all sets of tested matrices (Table 2).

In colostrum and PF, the mean concentration of IL-8 exceeded IL-4 (Fig. 2). The concentrations of IL-8 significantly differ between PF and sera of male (*p* = 0.00) and female (*p* = 0.00) piglets (Fig. 2E). No significant differences between IL-8 concentration in the sera of piglets belonging to opposite genders (*p* = 0.89; Fig. 2E) were found. Several correlations regarding the concentration of IL-8 in different matrices were identified (Table 2). Detected correlations concerning IL-8 concentration between different matrices were the weakest out of all analysed cytokines (Table 2).

In each type of examined matrix, except for PF, INF-γ was the most abundant cytokine (Fig. 2C). No significant differences between the concentration of INF-γ in the PF and the sera of male (*p* = 0.12) and female (*p* = 0.11) piglets were observed (Fig. 2C). No differences were identified between INF-γ levels in the serum of male and female piglets (*p* = 0.77; Fig. 2C). Significant, positive correlations between every tested set of matrices were demonstrated for this cytokine (Table 2).

Due to the undetectable concentration of TNF-α in three out of five tested matrices, further statistical analyses of this parameter were infeasible (Fig. 2F).

**Table 2:** The correlations (R-Spearman coefficient) in the concentration of IL-1β, IL-4, IL-6, IL-8, IFN-γ, and TNF-α between tested matrices. Brackets contain *p*-values for each correlation

	Cytokines									
	IL-1β		IL-4		IL-6		IL-8		INF-γ	
Matrices	Sows’ serum	Colostrum	Sows’ serum	Colostrum	Sows’ serum	Colostrum	Sows’ serum	Colostrum	Sows’ serum	Colostrum
Colostrum	0.89* (*p* = 0.00)	**1**	0.86* (*p* = 0.00)	**1**	0.78* (*p* = 0.00)	**1**	0.09 (*p* = 0.62)	**1**	0.84* (*p* = 0.00)	**1**
Male piglets’ serum	0.75* (*p* = 0.00)	0.80* (*p* = 0.00)	0.71* (*p* = 0.00)	0.79* (*p* = 0.00)	0.65* (*p* = 0.00)	0.67* (*p* = 0.00)	0.18* (*p* = 0.03)	-0.23* (*p* = 0.01)	0.61* (*p* = 0.00)	0.81* (*p* = 0.00)
Processing fluid	0.76* (*p* = 0.00)	0.78* (*p* = 0.00)	0.74* (*p* = 0.00)	0.79* (*p* = 0.00)	0.57* (*p* = 0.00)	0.62* (*p* = 0.00)	0.20* (*p* = 0.02	-0.10 (*p* = 0.24)	0.57* (*p* = 0.00)	0.76* (*p* = 0.00)
Female piglets’ serum	0.70* (*p* = 0.00)	0.76* (*p* = 0.00)	0.87* (*p* = 0.00)	0.91* (*p* = 0.00)	0.72* (*p* = 0.00)	0.69* (*p* = 0.00)	0.20 (*p* = 0.07)	-0.42* (*p* = 0.00)	0.78* (*p* = 0.00)	0.89* (*p* = 0.00)

*Correlation is significant (*p* < 0.05)

### Acute phase proteins

The concentration range of tested acute phase proteins (respectively for Hp (mg/mL), Pig-MAP (µg/ml) and CRP (µg/ml ) was as follow: for sows’ serum: 0.79–2.29, 484.71-1391.8, and 7.89–61.49, for male piglets’ serum: 0.29–1.56, 379.9–1471.00, and 4,69-29.62, for female piglets’ serum: 0.25–8.81, 372.97-1537.3, and 5.25–38.11, for PF 0.06–1.41, 372.49-1060.6, and 4.72–25.59, for colostrum 1.56–3.89, 457.4-1265.2, and 8.97–66.41.

**Fig. 3:**
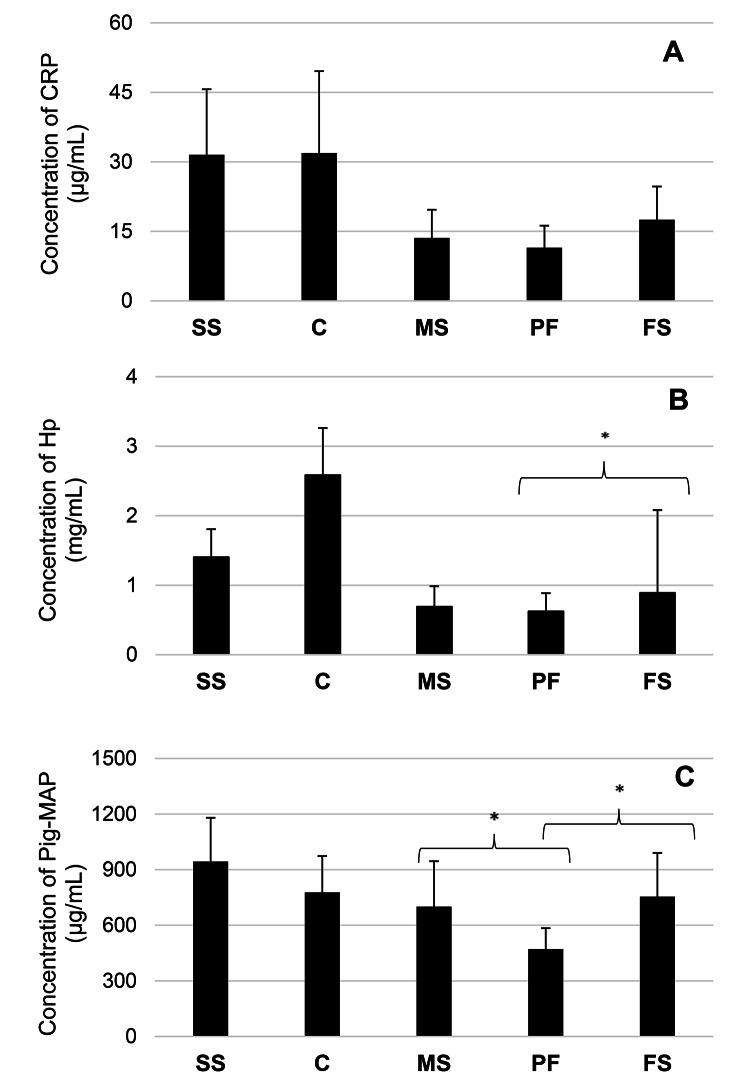
(**A-C**) The mean concentration (± SD) (mg/ml) of particular acute phase proteins: CRP, Hp, Pig-MAP in tested samples. SS – sows serum, C – colostrum, MS – male piglets’ serum, PF – processing fluid, FS – female piglets’ serum; * - statistically significant differences

Among analysed APPs, CRP had the lowest abundance in each tested matrix (Fig. 3). The concentration of CRP reached the highest concentration in sows’ serum and colostrum. In contrast, the lowest was in the PF (Fig. 3A). Statistically significant differences concerning CRP concentration were detected between the PF and sera of male (*p* = 0.00) and female (*p* = 0.00) piglets (Fig. 3A). Such differences were also demonstrated between the sera of male and female piglets (*p* = 0.00; Fig. 3A). Statistically significant correlations in the level of this parameter were only detected between sows’ serum and colostrum and between sera collected from sows and female piglets (Table 3).

Hp reached the highest concentration in each type of tested matrices, except for male piglets’ sera (Fig. 3B). There were no statistically significant differences between the concentration of Hp in the sera collected from piglets of different genders (*p* = 0.32; Fig. 3B). Also, the concentration of Hp in the sera of male piglets and PF did not differ significantly (*p* = 0.12; Fig. 3B). However, significant differences were found in the case of sera obtained from female piglets and PF (*p* = 0.02; Fig. 3B). Several positive correlations between Hp concentrations in different matrices were found (Table 3).

Pig-MAP was the abundant APP of male piglets’ serum (Fig. 3). In the remaining matrices, its mean concentration was second to the Hp (Fig. 3). The mean concentration of Pig-MAP in PF significantly differed from those measured in male (*p* = 0.00) and female (*p* = 0.00) piglets’ serum (Fig. 3C). At the same time, differences were not observed between the sera of piglets belonging to opposite genders (*p* = 0.06; Fig. 3C). A statistically significant correlation between all tested sets of matrices was observed (Table 3).

The concentration of SAA in most tested samples was below the test’s detection limit (0.31 µg/ml). SAA levels above the detection limit were found only in colostrum, with a mean concentration of 13.17 µg/ml (± 4.04). Therefore, any statistical analyses of this parameter were infeasible to perform.

**Table 3:** The correlations (R-Spearman coefficient) in CRP, haptoglobin, and Pig-MAP concentration between tested matrices. Brackets contain *p*-values for each correlation

	Acute phase proteins					
	CRP		Hp		Pig-MAP	
Matrices	Sows’ serum	Colostrum	Sows’ serum	Colostrum	Sows’ serum	Colostrum
Colostrum	0.78* (*p* = 0.00)	**1**	0.45* (*p* = 0.01)	**1**	0.4* (*p* = 0.03)	**1**
Male piglets’ serum	0.02 (*p* = 0.82)	0.04 (*p* = 0.63)	0.02 (*p* = 0.84)	0.17* (*p* = 0.04)	0.36* (*p* = 0.00)	0.57* (*p* = 0.00)
Processing fluid	0.09 (*p* = 0.26)	0.11 (*p* = 0.2)	0.08 (*p* = 0.32)	0.16* (*p* = 0.047)	0.09 (*p* = 0.23)	0.32* (*p* = 0.00)
Female piglets’ serum	0.26* (*p* = 0.01)	0.11 (*p* = 0.32)	0.05 (*p* = 0.65)	0.37* (*p* = 0.00)	0.23* (*p* = 0.03)	0.42* (*p* = 0.00)

*Correlation is significant (*p* < 0.05)

## Discussion

Blood serum is the most routinely used sample to monitor pig immune status. Due to some disadvantages in its collection, alternative matrices are desirable. As castration is still routinely performed on numerous farms, PF obtained during these procedures piqued scientists’ interest as a possible alternative. The results of several studies indicate that PF can be implemented in the monitoring of some pigs’ diseases. The usefulness of PF regarding the surveillance of swine diseases was initially assessed for PRRS [5]. The results of several studies showed that PF can be an efficient, cost- and work-saving alternative sample for PRRS monitoring in breeding herds [5, 9, 10]. The available data indicate that PF can also be applicable in PCV-2, PED, or Mhp monitoring [7, 8, 11]. Therefore, we hypothesised that the PF can be successfully employed for more diagnostics purposes, such as early assessment of porcine neonates’ immunological status.

Due to the epitheliochorial structure of the porcine placenta, the colostrum represents the sole source of passive immunity for piglets; the concentration of IgG in the piglets’ plasma at the age of 24 h is strongly correlated with colostrum intake [2]. Ig are crucial in conferring protection to piglets by the time they develop their immunity, so from an immunological point of view, they are the essential component of porcine colostrum. In the present study, the concentration of IgG, IgM, and IgA in various matrices, including PF, were evaluated, and correlations and differences between these concentrations were analysed. The results were consistent with previous studies in which IgG is the predominant Ig of porcine colostrum. It was followed by IgA and IgM, the least concentrated ones [3, 12–14]. The observed patterns of the mean concentration of tested Ig in sows’ sera, where IgG and IgA are the most and least concentrated, are also consensual with previously obtained results [15]. Consistent with previous studies, IgG was also the dominant Ig of piglets’ sera [3, 14]. Bandrick et al. (2014) showed that concentrations of IgG and IgA were higher in colostrum than in sows’ serum [16]. In the present study, colostrum contained a higher concentration of IgA. However, a greater concentration of IgG was determined in sows’ serum. Such differences can result from the timing of colostrum collection. In the present study, colostrum was obtained from 12 to 24 h from the start of parturition. In the study of Bandrick et al. (2014), it was collected within 1 h from the beginning of farrowing [16]. It is well documented that the concentration of Ig in colostrum declines rapidly during the first 24 h after farrowing, with the most marked decrease in the level of IgG [12, 14]. The decrease in the level of IgA is less drastic compared to IgG, and IgA is the predominant Ig of porcine milk [12, 14]. Moreover, more than 50% of IgA originates from the mammary gland; meanwhile, almost 100% of IgG is of serum origin [6]. The most prevalent Ig of PF was IgG, which IgA followed. The present study did not determine statistically significant differences regarding the concentration of IgG and IgA in the sera collected from piglets belonging to both genders and in PF. The results suggest that PF can be a reliable alternative to piglet serum for assessing the level of IgG and IgA. A slight positive correlation between the amount of IgA in the PF and the sows’ serum implies that PF can potentially be a helpful matrix in assessing the sows’ status regarding this Ig (R-Spearman = 0.25; *p* < 0.05). This statement, however, needs more studies on a larger population of animals. No correlation between the level of IgA in the PF, as well as serum collected from both genders of piglets vs. colostrum, was observed, which is interesting because most of the colostral IgA is produced locally in the mammary gland, and a lesser part is of serum origin [6]. This phenomenon may result from different times of taking samples. The correlation between PF and sows’ serum was not detected for IgG, almost wholly derived from sows’ serum [6]. We observed a moderate positive correlation between the level of IgA in the serum of sows and their colostrum. On the other hand, a study by Markowska-Daniel et al. (2010) did not show any correlation between IgA levels in these matrices [14]. The mentioned differences can result from the timing of sample collection. In the quoted research, the blood was sampled from sows 10 and 3 days before parturition, while in the present study, during 12–24 h after start of parturition [14]. No correlation between the concentration of IgG in sows’ serum and colostrum was detected, which aligns with previous results [14]. The lack of such correlation regarding IgG could result from the transfer of IgG from serum to colostrum, which depends not only on the IgG concentration in sows’ serum but also on some other factors, including season, genotype, vaccination, or earlier transfer of IgG from sows’ serum to colostrum, as the tight junctions between mammary gland cells are already open during the last month of gestation and enable the extensive transfer of Ig [14, 17]. In the present study, we observed high SD values concerning the mean concentration of IgG in sows’ serum and colostrum and IgA in colostrum, which resulted from high individual variability and could also influence statistical results. According to Bandrick et al. (2014), piglets’ serum concentration of IgG and IgA after ingestion of colostrum mimicked the distribution of these immunoglobulins in the sow’s colostrum [16]. The results of our study did not show a significant correlation between the concentration of IgG and IgA in the sera of piglets and colostrum, which is consensual with some previous results [14, 18]. The lack of such a relation is thought to be due to factors that can affect Ig concentration in the piglets’ sera, including the size of the litter, birth order, and gut closure timing [14]. On the other hand, another study showed a clear correlation between IgG concentration in these two matrices [19]. Nevertheless, this relationship was observed between colostrum obtained after the expulsion of the first piglet and before the first suckling and sera collected from piglets one-day following farrowing [19]. Concerning IgM, statistically significant differences were found between its amount in PF and serum obtained from piglets of both genders. The above implies that PF is unlikely an alternative to serum in the early assessment of IgM concentration in piglets. Of note, IgM is the least concentrated Ig of porcine colostrum. Therefore, in the early assessment of porcine neonate immune status, IgG and IgA seem of greater relevance. PF might be thus considered an alternative matrix for piglet IgG and IgA concentration assessment.

Multiple cytokines were detected in the porcine colostrum [3, 20, 21]. Similarly to the Ig, most cytokines are delivered for offspring via colostrum. It is thought that they play an instructive role in the maturation process of neonatal immunity [20]. Our results regarding the concentration of particular cytokines in the sows’ serum and colostrum differ from previous research in which the most abundant cytokine in the porcine colostrum and sows’ serum was IL-4 [20]. In the present study, the most abundant cytokines in these two matrices were IFN-γ, IL-1β, and IL6; meanwhile, IL-4 was one of the least concentrated ones in the tested matrices. As IFN-γ, IL-1β, and IL6 are classified as pro-inflammatory cytokines, such differences can indicate and result from some ongoing inflammation processes. However, the differences between the IL-6 amounts in these two studies were not enormous, while in the study of Nguyen et al. (2007), the concentration of IL-1β was not assessed [20]. Moreover, in the present research, TNF-α, also associated with inflammation, was detected in the sows’ serum but was absent in the study of Nguyen et al. (2007) [20]. Nguyen et al. (2007) hypothesise that TNF-α present in colostrum is produced locally in the mammary gland and does not originate from sows’ circulation [20]. In the study of Maciag et al. (2022), the predominant cytokine of sows’ serum was INF-γ, consistent with our results [3]. In the quoted study, the highest concentrated cytokine of sows’ colostrum was IL-4, which, on the other hand, is in line with the results obtained by Nguyen et al. (2007) [3, 20]. The content of cytokines in piglets’ sera detected in our study was also different compared to the study of Nguyen et al. (2007). Contrary to our research, in which IFN-γ, IL-1β, and IL-6 reached the highest concentration in piglets’ sera, in the quoted study, the most abundant cytokine of suckling piglets’ serum was IL-4, which was followed by TGF-β [20]. Piglets do not produce endogenously other cytokines, except IL-12 and TGF-β1 [20]. Additionally, cytokines do not cross the placenta [20]. Therefore, observed differences seem understandable, as colostrum constitutes the sole source of particular cytokines [20]. In the study of Maciag et al. (2022), the most concentrated cytokine of piglets’ sera was IFN-γ, followed by IL-4 [3]. The most significant differences compared to our results were presented in the Llamas Moya et al. (2007) study, in which the piglets’ plasma concentration of IL-1β was much lower compared to our results (< 290 pg/ml) [22]. Moreover, the presence of TNF-α was detected in the piglets’ sera [22]. The minimum and maximum concentrations of TNF-α were observed at one day and five days of age, respectively [22]. Of note, neither the present nor any other results detect TNF-α in the piglets’ serum [20]. No significant differences regarding the concentration of IL-1β, IL-4, IL-6, and IFN-γ between PF and piglets sera and between sera collected from piglets of both genders were found in the present study. The above implies that PF can represent a promising alternative for blood in assessing these cytokine concentrations in neonatal piglets. Similar results were reported in the previous study, where the correlation in the concentrations of IFN-γ, IL-4, and IL-6 in sows’ serum and colostrum has been noted [20]. Therefore, concerning these cytokines, it can be assumed that its sows’ serum concentrations contribute to its level of mammary secretion [20]. Regarding sows’ serum and colostrum, we have also observed a positive correlation of IL-1β concentration. Of note, its content was higher in colostrum compared to serum. Such correlation was not found for IL-8 concentration. Moreover, IL-8 content was higher in the colostrum compared to the sows’ serum, suggesting that this cytokine does not originate solely from serum but can be produced locally in the mammary gland [20]. These findings, however, need further confirmation. Notably, the observed lack of correlations may result from cytokines’ relatively short half-life and instability [20, 23]. The concentration of IL-8 in colostrum was negatively correlated with its level in piglet sera. The study performed on human colostrum indicates that cytokines in colostrum lose their stability depending on the combination of time and the temperature in which it is stored [24]. Concerning INF-γ, IL-β, IL-4, and IL-6 concentrations, positive correlations were found between sera collected from piglets and sows and between piglets’ sera and colostrum. Therefore, according to Nguyen et al. (2007) results, it can be assumed that piglets’ serum concentration of these cytokines corresponds to the mother pattern [20]. Significant, strong correlations were found between the concentration of IL-1β, IL-4, IL-6, and IFN-γ in PF and sows’ serum and between IL-1β, IL-4, IL-6, and IFN-γ in PF and colostrum. This indicates that PF can be potentially useful in indirectly assessing mentioned cytokines. However, it should be in mind that regarding the concentrations of most tested cytokines, relatively high SD values were observed, which could affect the results of statistical analyses and influence the significance of observed results (Fig. 2).

Hp, Pig-MAP, SAA, and CRP are classified as positive, major pigs’ APP [25]. They are produced and released due to pro-inflammatory cytokines activation in response to inflammation, which can be caused by infections, stress, or tissue injuries [26]. Therefore, APP can be a valuable indicator of health status disruption. Available data indicate an increased interest in APP as a health status marker that can be routinely used in pig production [26]. Concerning APP during the early stages of piglets’ life, the literature data is less abundant than that of Ig. In line with several previous studies, the presence of Hp, Pig-MAP, and CRP was confirmed in colostrum and piglets’ sera [27–29].

The mean concentration of CRP in the serum of piglets observed in the present study was similar to previously observed (17.51 µg/ml in female piglets, 13.59 µg/ml in male piglets vs. ~15 µg/ml) [22]. The amount of CRP in the piglets’ serum does not vary with age within the first week of life [22]. It is well documented that the plasma CRP concentration increases rapidly after the inflammatory stimulus [30]. Herein, significant differences were determined between the concentration of CRP in piglets’ serum of both genders and between piglets’ serum and PF. Therefore, PF should not be considered a reliable matrix for assessing piglets’ CRP concentration. Interestingly, a positive correlation between the amount of CRP was demonstrated for sera and saliva [31]. The present study detected no significant correlation between the amount of CRP in the colostrum and piglets’ sera, which agrees with the previous results [29]. The amount of CRP in both matrices observed in the present study was much higher than the quoted one [29]. It is considered that the piglets’ serum CRP content originates from colostrum at the 4 days of life [29].

The concentration of Hp in particular matrices was similar to those documented in the previous study, in which its content in the colostrum and sows’ serum was 0.78–1.11 mg/ml and 2.58 mg/ml, respectively [28]. We observed, however, more significant differences in the concentration of Hp in the piglets’ serum compared to a previous study, in which its concentration at 9 h post-partum in the serum of piglets that ingested colostrum was 271 ± 48.6 µg/ml [28]. Such differences can result from blood collection timing. It was demonstrated that the concentration of plasma Hp in piglets increases within the first week of life [22, 27]. In the second experiment described in the quoted research, Hp concentration was measured in the sera collected from piglets at 14 days and during weaning. The obtained results were much more comparable to ours (0.58 ± 0.04 mg/ml and 0.53 ± 0.04 mg/ml on the 14th day and at the weaning, respectively) [28]. Concerning Hp piglets’ serum content, our results were different from those presented by Llamas Moya et al., in which the Hp amount in the piglets’ plasma ranged from 0.6 to 1.2 ng/ml on the 3rd and 5th day of life, respectively [22]. It is worth adding that the observed differences may result from different designs of both experiments (commercial farm vs. controlled conditions), as environmental factors (dust, gases) influence the level of Hp [32]. Hp is of colostral origin [28].

Moreover, colostrum increases its piglets’ endogenous production [28]. A positive correlation between the Hp amount in colostrum and piglets’ serum observed in the present study is consistent with the above finding. We also found a positive correlation between colostrum and sows’ serum, which may indicate that the level of Hp in the colostrum depends on its level in the sows’ blood. However, such a relationship was not determined between the sera of sows and their offspring or sows’ serum and PF. Positive correlations of Hp levels were also observed in other matrices, like serum and meat juice, and between serum and saliva [26, 31]. Despite the lack of statistically significant differences between the amount of Hp in sera collected from piglets of both genders, we confirmed such differences concerning PF and female piglets’ sera. These findings can result from more pronounced inter-female piglet variability regarding Hp content in female sera. Therefore, PF is unsuitable for determining the Hp content in piglets.

The data concerning Pig-MAP during the perinatal period of suckling piglets needs to be revised. Using a Western blot technique, Martin et al. (2005) have determined, among others, the presence of 120 kD bands in colostrum and piglets’ serum corresponding to the native Pig-MAP/ITIH4 protein [27]. Based on these findings, the authors suggested that colostrum could be, at least in part, a source of piglets’ blood Pig-MAP [27]. We have detected Pig-MAP’s presence in each tested specimen, including colostrum and piglets’ sera. Moreover, we have determined a positive correlation between the Pig-MAP amount in colostrum and piglets’ sera. Therefore, the present results confirm the observation of Martin et al. (2005). A positive correlation in the current study between sows’ serum and colostrum may indicate that colostrum Pig-MAP is of serum origin.

Furthermore, a significant relationship between sows’ and piglets’ serum suggests that the concentration of this APP in the sows’ circulation influences its concentration in piglets. Concerning other alternatives to serum matrices, a positive correlation in the content of Pig-MAP was found between meat juice and serum [26]. The Pig-MAP concentration in PF significantly differed from the concentration measured in the piglets’ serum of both genders. Of note, the SD values regarding Pig-MAP in this study were high, which could affect the results of statistical analyses. Therefore, the utility of PF for assessing the concentration of Pig-MAP requires further research.

## Conclusion

To the authors’ knowledge, this is the first report in which the presence of Ig, cytokines, and APP was confirmed in PF. Due to the lack of significant differences between the concentration of IgG, IgA, IL-1β, IL-4, IL-6, and IFN-γ in the serum of piglets and PF, it can be assumed that PF represents a promising alternative to blood for their assessment in suckling piglets. Moreover, due to correlations between IgA, IL-1β, IL-4, IL-6, IL-8, IFN-γ concentrations in sows’ serum and PF and Pig-MAP concentrations in colostrum and PF, PF can potentially represent a valuable and relevant matrix for the indirect assessment of sows’ immune parameters. Since this is the first study evaluating the PF as a potential matrix for evaluating piglets’ and sows’ immune status, further analyses are desirable.

## Methods

### Animals

The study was conducted on a farrow-to-finish pig herd of 100 sows (Danbred hybrid). The animals were privately owned by the farmer. Samples were collected from 264 pigs (31 sows, 146 male piglets, and 87 female piglets). Lots of 9–10 sows were formed every 21 d. Sows and suckling piglets were kept in a well-prepared farrowing room. There was no cross-fostering of piglets within piglets in this study. An all-in/all-out swine production system with thorough cleaning and disinfection in between lots was implemented routinely on this farm. Standard farm management included castration and tail docking at 3–5 days and weaning at 4 weeks of age. Only sows between 2 and 4 parities, weighed 150–200 kg, and farrowing without assistance, at least 10 piglets were included in this study. The herd’s routine health monitoring program included serological profiling and colostrum quality analysis.

Based on serology test results, the herd was confirmed seronegative to pseudorabies virus (PRV), PRRSV, influenza A virus (IAV), *Actinobacilus pleuropneumoniae* (App), and *Mycoplasma hyopneumoniae* (Mhp). Clinical, and anatomopathological examinations (regularly performed ante-mortem inspection of carcass at slaughterhouse) showed no evidence of streptococcosis, pleuropneumonia, Glasser’s disease, or atrophic rhinitis. The breeding sows were vaccinated against porcine parvoviral infection, erysipelas, atrophic rhinitis and colibacillosis of newborn piglets.

### Samples collection and processing

Thirty-one colostrum samples, one from each sow, were collected between 12 and 24 h after the start of farrowing. Therefore, regarding sampling time, the analysed colostrum was middle-late, which could influence the concentration of measured indices and obtained results. All samples were transported to a laboratory in a cooler condition and then refrigerated until further analyses. Blood was collected from the *vena jugularis* or *vena cava cranialis* into clot activator tubes. Blood from sows was collected simultaneously to colostrum (12–24 h after the farrowing onset). Sampling of piglets took place 2–5 days after farrowing (at the time of piglets processing). Samples were collected from at least seven piglets from each litter. A total of 31 sows’ serum samples, 146 male serum and PF samples, and 87 female serum samples were included in the present study. Blood was centrifuged (2500 x g, 15 min, 4^o^C) to obtain serum and kept frozen (-80^o^C) until further analyses. After the experiment, the pigs were released for rearing.

### Laboratory analyses

IgA, IgG, and IgM concentrations in serum, colostrum, and PF were analysed using species-specific commercial ELISA kits (Porcine Immunoglobulin G, IgG ELISA Kit, Porcine Immunoglobulin A, IgA ELISA Kit, Porcine Immunoglobulin M, IgM ELISA Kit) from BT Laboratory (Jiaxing, Zhejiang, China). All tests were conducted according to the manufacturer’s recommendations. Table 4 presents the dilution of each sample type used in the present study. The concentration of each parameter was calculated based on a standard curve for each using Magellan v.7.2 software (Tecan). For all kits, the intra- and interassay CV were < 8% and < 10%, respectively. Detection limits were 0.022 mg/ml for IgM, 0.251 mg/ml for IgG, and 10.25 µg/ml for IgA.

The concentration of the following cytokines: TNF-α, IFN-γ, IL-1β, IL-4, IL-6, and IL-8 in serum, colostrum, and PF was determined with the use of commercial species-specific ELISA kits (RayBio® Porcine TNF-alpha ELISA Kit, RayBio® Porcine IFN-gamma ELISA Kit, RayBio® Porcine IL-1 beta ELISA Kit, RayBio® Porcine IL-4 ELISA Kit, RayBio® Porcine IL-6 ELISA Kit, RayBio® Porcine IL-8 ELISA Kit) from RayBiotch Inc (Norcross, GA, USA), following the manufacturer procedures. The concentration of analysed parameters was calculated based on a standard curve for each using Magellan v.7.2 software (Tecan). The intra- and interassay CV were < 10% and < 12% respectively. The minimum detectable concentration of cytokines were 6pg/ml (IL-1beta); 1.5pg/ml (IL-4), 45pg/ml (IL-6); 10pg/ml (IL-8), 20 pg/ml (TNF-α) and 500pg/ml (INF-γ).

The concentration of SAA, CRP, and Hp in serum, colostrum, and PF was determined with the use of commercial ELISA kits (Pig serum amyloid A ELISA, Pig haptoglobin ELISA, Pig C-reactive protein (CRP) ELISA) from Life Diagnostics Inc. (West Chester, PA, USA). Pig-MAP concentration in all matrices was determined with the ELISA kits (Acuvet ELISA PigMAP) from Acuvet Biotech SL (Zaragoza, Spain). The intra- and interassay CV for all Elisa assays used in this study were < 10% and < 12% respectively. Detection limits of test used were: 4,68 µg/mL for CRP, 0,05 mg/mL for Hp, 0,31 µg/ml for SAA and 0.18 µg/mL Pig-MAP. Inter and intra assays CV of the kits used in the study are lower than 10%. All tests were conducted according to the manufacturer’s recommendations. The concentration of analysed parameters was calculated based on a standard curve for each using Magellan v.7.2 software (Tecan).

To ensure the precision of results, all samples were run in duplicate, and results were considered acceptable when the relative standard deviation was ≤ 10%. Repeatability and reproducibility ELISA tests used in our study performed at 3 concentration levels (if available) of analytes in PF and colostrum (negative samples, low and high concentration) yielded values below 9.5% CV for repeatability and below 11.5% CV for reproducibility. Accuracy was investigated by linearity under dilution; in brief, two PF and colostrum samples were diluted (1:2; 1:4; 1:8; 1:16, 1:32) with sample diluents. Dilution studies resulted in linear regression equations with a correlation coefficient ranged from 0.97 to 0.99 showing that the tests measures the proteins in a linear manner.

**Table 4:** Samples dilutions depending on the expected analyte concentration and the manufacturer’s recommendation

Parameter	Sample	Dilution Factor
IgA	Serum, PF	No dilution
	colostrum	1:100
IgG	Serum, PF	No dilution
	colostrum	1:100
IgM	Serum, PF	No dilution
	colostrum	1:100
IFN-γ	Serum, PF, colostrum	1:2
TNF - α	Serum, PF, colostrum	1:2
IL-8	Serum, PF, colostrum	1:2
IL-6	Serum, PF, colostrum	1:2
IL-1β	Serum, PF, colostrum	1:2
IL-4	Serum, PF, colostrum	1:2
Pig-MAP	Serum, colostrum	1:1000
	PF	1:100
Hp	Serum, PF, colostrum	1:10 000
CRP	Serum, PF, colostrum	1:2000
SAA	Serum, PF, colostrum	1:400

### Statistical analysis

All the data were analysed using Statistica 13.3 (Tibco, USA). The significance level was α = 0.05, and a *p*-value < 0.05 was considered statistically significant. The obtained data were subjected to the W. Shapiro-Wilk test for normality and Levene’s test for equality of variances. Due to the nonparametric distribution of the analysed variables, Spearmans’ rank correlation coefficient test was used to determine the correlation between various matrices, i.e. sows’ serum and piglets’ serum, sows’ serum and colostrum, piglets’ serum and colostrum, sows’ serum and PF, piglets’ serum and PF, or colostrum and PF. Differences between mean concentrations of investigated parameters in PF vs. piglets serum and serum of females vs. serum of males were tested by a nonparametric U Mann-Whitney test.

## Data Availability

The data used to support the findings of this study are available from the corresponding author upon reasonable request.

## Abbreviations

APP: Acute phase proteins
CRP: C – reactive protein
Hp: Haptoglobin
IFN: Interferon
IL: Interleukin
Ig: Immunoglobulins
PF: Processing fluid
TNF: Tumour necrosis factor
